# Temporal variability is a personalized feature of the human microbiome

**DOI:** 10.1186/s13059-014-0531-y

**Published:** 2014-12-03

**Authors:** Gilberto E Flores, J Gregory Caporaso, Jessica B Henley, Jai Ram Rideout, Daniel Domogala, John Chase, Jonathan W Leff, Yoshiki Vázquez-Baeza, Antonio Gonzalez, Rob Knight, Robert R Dunn, Noah Fierer

**Affiliations:** Department of Biology, California State University, Northridge, Northridge, CA 91330-8303 USA; Department of Biological Sciences, Northern Arizona University, Flagstaff, AZ 86011 USA; Center for Microbial Genetics and Genomics, Northern Arizona University, Flagstaff, AZ 86011 USA; Cooperative Institute for Research in Environmental Sciences, University of Colorado, Boulder, CO 80309 USA; Department of Genetics and Genomic Sciences, Icahn School of Medicine at Mount Sinai, New York, NY 10029 USA; Department of Ecology and Evolutionary Biology, University of Colorado, Boulder, CO 80309 USA; Department of Computer Science, University of Colorado, Boulder, CO 80309 USA; BioFrontiers Institute, University of Colorado, Boulder, CO 80309 USA; Department of Chemistry and Biochemistry, University of Colorado, Boulder, CO 80309 USA; Howard Hughes Medical Institute, University of Colorado, Boulder, CO 80309 USA; Department of Biological Sciences and Keck Center for Behavioral Biology, North Carolina State University, Raleigh, NC 27607 USA

## Abstract

**Background:**

It is now apparent that the complex microbial communities found on and in the human body vary across individuals. What has largely been missing from previous studies is an understanding of how these communities vary over time within individuals. To the extent to which it has been considered, it is often assumed that temporal variability is negligible for healthy adults. Here we address this gap in understanding by profiling the forehead, gut (fecal), palm, and tongue microbial communities in 85 adults, weekly over 3 months.

**Results:**

We found that skin (forehead and palm) varied most in the number of taxa present, whereas gut and tongue communities varied more in the relative abundances of taxa. Within each body habitat, there was a wide range of temporal variability across the study population, with some individuals harboring more variable communities than others. The best predictor of these differences in variability across individuals was microbial diversity; individuals with more diverse gut or tongue communities were more stable in composition than individuals with less diverse communities.

**Conclusions:**

Longitudinal sampling of a relatively large number of individuals allowed us to observe high levels of temporal variability in both diversity and community structure in all body habitats studied. These findings suggest that temporal dynamics may need to be considered when attempting to link changes in microbiome structure to changes in health status. Furthermore, our findings show that, not only is the composition of an individual’s microbiome highly personalized, but their degree of temporal variability is also a personalized feature.

**Electronic supplementary material:**

The online version of this article (doi:10.1186/s13059-014-0531-y) contains supplementary material, which is available to authorized users.

## Background

The increasing recognition that commensal and mutualistic microorganisms are necessary for many aspects of normal human physiology has altered the traditional pathogen-dominated view of human-bacterial interactions [1,2]. As a result of this paradigm shift, there is a tremendous amount of interest in understanding the factors that influence the diversity, composition, dynamics, and function of human-associated microbial communities. One of the primary objectives is to leverage this understanding in order to manage, restore, and/or exploit our microbial partners in ways that promote human health. However, our current understanding of how and why these communities vary through time is limited. Previous studies that have characterized human associated microbial communities over time have been based on relatively few individuals [3,4], intermittent sampling intervals [2,5,6], single body habitats [4,7-10], or focused on disease states [11], leaving us with an incomplete picture of the range of normal variability in the human microbiome. Only by conducting longitudinal studies of large cohorts of both healthy and diseased hosts can we begin to identify the ecological factors structuring the diversity, composition, and dynamics of the human microbiome.

Here, we investigated the temporal dynamics of forehead, gut (feces), palm, and tongue microbial communities of 85 college-age adults (Table 1) from three U.S. universities. Samples were self-collected weekly over a 3-month period beginning in January 2012. Bacterial and archaeal communities were characterized using high-throughput sequencing of the variable region 4 (V4) of the 16S rRNA gene [12]. In total, we generated 170,563,932 quality-filtered sequences from 3,655 samples, with all analyses conducted on samples rarefied to exactly 10,000 sequences per sample. To identify potential drivers of variability, we collected demographic, lifestyle, and hygiene data at the initiation of the sampling period using a standardized 49-question survey (Additional file 1). Weekly questionnaires were used to track changes in health status, medication use, menstrual cycle for women, and other dramatic changes in routine behavior (Additional file 2). De-identified responses to all questions are provided in Additional file 3.

**Table 1:** **Demographic summary of study participants**

**Subject ID** ^**a**^	**Age (years)**	**Gender**	**BMI**	**Ethnicity**	**Samples (forehead/gut/palm/tongue) (n)**
A000	21	Female	19.94	Caucasian	9/9/10/10
A003	21	Female	18.56	Caucasian	10/10/10/10
A004	22	Female	25.85	Caucasian	8/10/10/10
A007	22	Female	19.97	Caucasian/Asian	9/9/8/9
A008	20	Male	20.67	Caucasian	10/10/9/-
A009	20	Female	22.31	Caucasian	9/9/9/10
A010	20	Female	?	Caucasian	10/10/8/10
A011	29	Female	19.46	Hispanic	7/8/7/8
A012	21	Female	26.61	Caucasian	10/9/10/10
A015	20	Female	24.13	Caucasian	9/9/9/9
A016	22	Female	25.75	Caucasian	8/-/7/9
A017	21	Female	33.84	Caucasian	9/9/9/9
A019	22	Female	18.29	Caucasian	9/9/9/9
A026	21	Male	22.24	Caucasian	8/8/8/8
A027	20	Female	21.79	Other	9/9/9/9
A028	23	Male	23.06	Asian/Pacific island	8/8/7/8
A029	21	Male	25.83	Caucasian	7/9/9/9
A032	21	Female	18.88	Caucasian	8/8/-/8
A033	21	Female	27.44	Caucasian	10/9/10/10
A036	20	Female	21.29	Caucasian	8/8/-/8
A037	20	Female	20.80	Hispanic	9/8/9/10
A038	21	Female	?	Hispanic	8/8/8/9
A040	22	Male	23.09	Caucasian	10/9/10/10
A042	36	Male	25.40	Caucasian	9/10/10/10
A044	21	Male	26.58	Caucasian	10/10/9/9
A048	22	Female	22.14	Caucasian	10/10/10/10
A049	20	Female	?	Caucasian	7/8/-/10
A050	41	Female	22.86	Caucasian	9/9/9/9
A051	20	Male	22.47	Caucasian	7/-/7/-
A052	32	Male	31.84	Hispanic	10/10/10/10
A053	23	Female	21.14	Caucasian	10/10/9/10
A056	23	Male	23.73	Caucasian	8/8/8/8
A061	?	Male	?	?	7/-/-/7
B101	24	Male	18.31	Caucasian	9/9/9/9
B102	32	?	?	caucasian	7/-/-/7
B105	?	?	?	caucasian	7/7/7/7
B106	21	Female	21.95	Caucasian	8/9/8/9
B107	19	Female	20.37	Caucasian	9/8/-/9
B108	30	Female	24.80	Asian/Pacific island	7/7/-/7
B109	24	Male	23.67	Caucasian	9/9/8/9
B110	20	Female	20.36	Caucasian	9/9/9/9
B114	21	Female	17.54	Caucasian	8/9/-/9
B117	?	Female	?	Caucasian	8/7/-/8
B119	20	Male	?	Caucasian	-/8/-/8
B121	20	Female	22.86	Caucasian	9/9/8/9
B123	20	Male	25.07	Caucasian	9/8/9/9
B124	21	Female	22.15	Caucasian	9/9/-/9
B129	21	Female	18.40	Caucasian	9/8/-/9
B130	22	Female	22.67	Caucasian	8/9/-/9
B132	22	Female	16.82	Hispanic	9/9/9/9
B133	22	Male	27.89	Caucasian	9/9/8/7
B134	?	Male	21.91	Caucasian	7/-/7/7
B136	22	Female	19.22	Caucasian	8/9/7/8
B137	22	Female	?	Hispanic	-/7/-/7
B139	20	Female	17.75	Caucasian	9/9/-/9
B144	33	Male	25.10	Caucasian	8/8/-/8
B146	26	Female	20.60	Caucasian	-/7/-/-
B147	51	Female	?	Caucasian	7/7/7/-
B148	37	Male	20.08	?	8/8/7/8
B149	55	Male	25.10	Caucasian	9/9/9/9
B150	32	Female	20.05	Caucasian/Hispanic	8/9/9/9
B153	21	Female	21.93	Caucasian	8/8/-/8
B154	21	Female	23.40	Caucasian	9/9/7/9
B155	30	Female	23.62	Caucasian	9/9/9/9
B156	?	Female	?	Hispanic	-/7/-/7
B157	25	Male	21.86	Caucasian	10/9/9/9
B159	21	Male	25.10	Caucasian	7/8/-/9
B160	22	Female	17.75	Asian/Pacific island	8/8/7/8
B161	21	Male	26.58	Caucasian	7/-/-/7
B164	22	Male	22.96	Caucasian	7/7/7/7
C203	23	Female	21.74	Caucasian	9/7/9/9
C204	25	Male	22.31	Caucasian	9/9/7/9
C210	20	Female	24.03	Caucasian	8/-/8/8
C212	22	Female	24.30	Caucasian	8/8/8/8
C213	18	Female	22.71	Caucasian	8/8/7/8
C214	27	Male	27.71	Caucasian	7/7/8/8
C233	19	Male	22.96	Caucasian/Hispanic	9/7/9/8
C234	18	Female	32.10	Caucasian	9/-/8/9
C236	18	Female	18.09	Caucasian	-/-/-/9
C237	19	Male	18.65	Caucasian	7/7/-/-
C243	23	Male	23.63	Caucasian	8/-/8/8
C248	21	Male	20.71	Caucasian	9/8/9/8
C253	27	Female	23.21	Caucasian	8/9/9/9
C255	22	Female	27.46	Caucasian	8/9/-/9
C263	20	Male	20.09	Caucasian	7/8/8/7

^a^Single-letter prefix represents the university of attendance.

Question marks denote data not provided by study participants. The last column shows the number of samples used in the time series analysis for each body habitat from each subject. Dashes indicate that samples from that individual were not including in the analysis for that particular body habitat. For full metadata, the reader is referred to Additional file 3.

BMI = body mass index.

## Results and discussion

**Figure 1:**
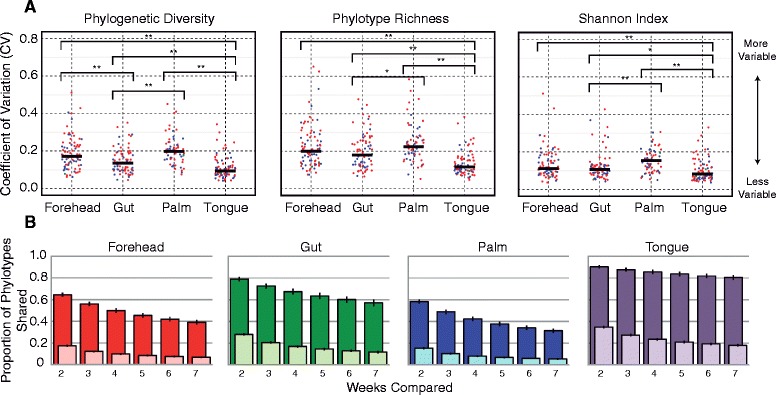
**Body habitats exhibited different levels of temporal variability both in diversity (A) and membership (B).** In (A), each point represents the temporal variability of a single individual colored by gender (red = female, blue = male) with black bars representing the median for a given body habitat and metric. Statistical differences were observed for each metric across body habitats (Kruskal-Wallis, *P* ≤0.01) and comparisons based on pairwise Mann-Whitney *U*-test are denoted by asterisks (* = corrected *P* ≤0.05, ** = corrected *P* ≤0.01). In (B), the smaller, lighter shaded bars in each plot are for all phylotypes except singletons and the larger, darker bars are only for the 100 most abundant phylotypes for each individual. Error bars in (B) are ±1 SEM.

To quantify the amount of temporal variability in diversity of each body habitat, we calculated the coefficient of variation (CV = standard deviation/mean) for three alpha diversity metrics (phylogenetic diversity, phylotype richness, and Shannon index [13]) for each individual [14]. Low CV values indicate that an individual had relatively stable alpha diversity levels, whereas high CV values indicate than an individual had variable levels of alpha diversity over the 3-month study period. As evident in Figure 1a, there was a wide range of variability within each body habitat indicating that some individuals varied more than others. When we compare values across body habitats, we see that skin surfaces, particularly the palm, exhibited higher levels of temporal variability in diversity than gut or tongue (Figure 1a). These patterns were generally consistent regardless of the alpha diversity metric used. Skin surfaces also hosted the most diverse communities we surveyed (Additional file 4), as theory would predict for open, exposed environments that have a larger species pool of potential colonizers [15]. Ecological theory also predicts that habitats with large species pools should vary more through time [15], which is what we observed here.

**Figure 2:**
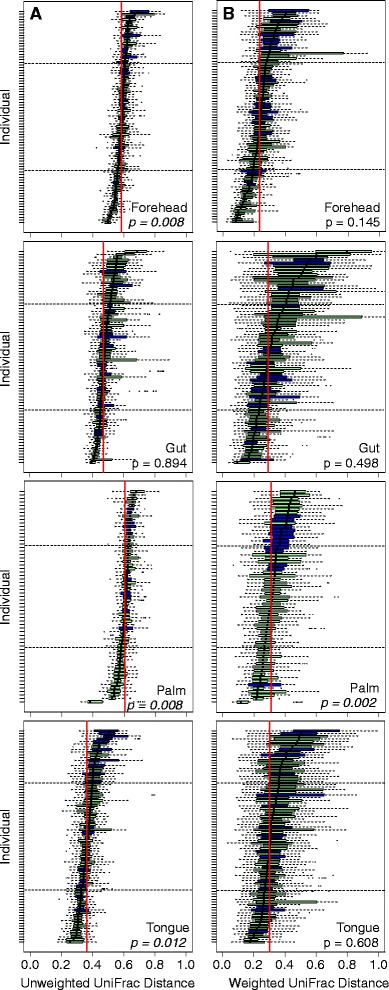
**Boxplots of unweighted (A) and weighted (B) intra-individual UniFrac distances for each body habitat.** A broad range of temporal variability in microbial community membership (A) and structure (B) was observed across body habitats and within body habitats across individuals. Individuals are sorted by median in each plot. Green bars depict individuals who did not report antibiotic use during the study period while blue bars indicate individuals who took antibiotics. The median values for each body habitat are shown with vertical red lines. Dotted horizontal lines in each plot divide the study population into first and fourth quartiles and depict ‘stable’ and ‘variable’ individuals, respectively. Non-parametric Mann-Whitney U-tests were used to determine the affect of antibiotic use on temporal variability within each body habitat. *P* values are shown in each panel. Note that statistical differences were observed for each metric across body habitats (Kruskal-Wallis, *P* ≤0.01).

This high degree of temporal variability in alpha diversity levels was matched by high variability (and hence instability) in community membership (Figure 1b). Comparing the proportion of phylotypes shared among time intervals within an individual shows that fewer phylotypes were shared through time in skin communities than in the gut or tongue communities. For example, on average only 15% of the phylotypes observed on the palm skin surface (excluding singletons on a per individual basis) were observed at any other point in time, whether samples were collected 1 or 6 weeks apart. A similar pattern was observed when we used median unweighted UniFrac values [16] (a phylogenetic metric of community membership) for each body habitat, where turnover was found to be greater for the skin than for the tongue and gut communities (Figure 2a and Additional file 5a). In contrast, variability in community structure, which accounts for phylotype abundance (median weighted UniFrac), was lower on the forehead than the other body habitats (Figure 2b and Additional file 5b), suggesting that the nature of variability differs depending on the body habitat in question. On the tongue and in the gut, changes in the relative abundance of persistent taxa (that is, those taxa that are consistently present over time) drive the temporal dynamics, whereas temporal variability in forehead communities appears to be driven more by the presence or absence of transient taxa on the skin surface. For the palm, both membership and structure appear highly dynamic, likely due to frequent hand washing and exchange of microbes with the numerous surfaces we touch on a daily basis, including our other body parts.

Using median UniFrac values for each individual as our metric of temporal variability in community membership (unweighted) and structure (weighted), we found that individuals differed dramatically not only in the composition of their microbial communities (Additional file 6), as has been observed previously [2,5,17,18], but also in the degree of temporal variability observed in their microbial communities (Figure 2 and Additional file 7). This has been previously shown in vaginal communities [8], but we show here that this is a general characteristic of microbial communities across human body habitats. The variability of microbial communities in one body habitat, in general, did not predict the variability of microbial communities of other body habitats. The exception was the two skin habitats, where individuals that had more variable forehead communities also had more variable palm communities (Additional files 8 and 9). This finding suggests that the factors that contribute to intra-personal temporal variability in microbiome composition are shared across skin habitats, but not necessarily across other body habitats. Furthermore, relatively few individuals exhibited a significant time-decay relationship [19]; in general, samples collected closer together in time did not harbor more similar communities than those collected further apart in time (Additional file 10). These results highlight that attempts to predict what type of communities to expect in a given body habitat based on data collected during the previous week (or weeks) may be difficult for most individuals. However, it is important to note that if we had sampled more frequently (for example, on a daily basis [3]) or for a longer period of time, we may have been able to identify a stronger relationship between elapsed time and the composition of the communities within body habitats.

Having established that the degree and nature of variability was specific to each body site and was in itself an important parameter that distinguished individuals from one another, we next sought to identify factors associated with this variation across individuals. Based on previous work [4,20], we expected that antibiotic usage would lead to profound shifts in the structure of an individual’s microbiome. Indeed, within a given individual, the largest shifts observed in community membership coincided with the time points that the individual reported having taken oral antibiotics (*P* <0.001 for both unweighted and weighted UniFrac, Monte Carlo *t*-test with 1,000 iterations). Across the study population, however, with the exception of palm communities, we did not find a significant effect of antibiotics on variability in community membership and structure; individuals who took antibiotics did not, on average, have more variable communities than those that did not take antibiotics over the time period of this experiment (Figure 2). Our observation that antibiotic use was not associated with increased temporal variability in microbial communities across the study population could be due to the fact that we did not control for the timing of sampling relative to antibiotic use, dosage, or type of antibiotics used by the individuals sampled here, or it may be because microbial community responses to antibiotics are highly individualized, as suggested by recent work [4,21].

We next used generalized linear models (GLMs) to identify which other factors or combination of factors best predicted why some individuals harbored more variable microbial communities than others. For these models, we again used median weighted or unweighted UniFrac values of each individual as our response variables for each body habitat. Potential predictive factors were compiled from the initial survey responses (Additional file 2) and we only included factors for which we had sufficient replication in survey responses (Additional file 3). Presented models included factors with a significance value <0.05. As shown in Table 2, our models were often able to explain much of the variability in the temporal stability of microbial communities across individuals, but the strength of the models was dependent on the body habitat in question or the distance metric used. Common predictive factors observed in multiple body habitats included median alpha diversity values (Shannon Index), university affiliation, and antibiotic use (Table 2). However, the strongest predictive variable for most body habitats was median diversity, measured using the Shannon index, suggesting an overall relationship between diversity and variability. Other factors appeared to have a body site-specific affect. For example, the number of roommates helped explain a significant amount of variability in the structure (weighted) of forehead microbial communities, a pattern that may driven by the exchange of skin bacteria between individuals sharing a common living area.

**Table 2:** **Measured factors that influenced the temporal variability of the human microbiome**

	**Parameter estimate**	**Sum of squares**	**F statistic**	***P*** **value**	**BIC**	**R** ^**2**^
*Forehead - unweighted*						
Antibiotic use	0.015	0.010	8.76	0.004	-262.21	0.175
University	0.119	0.006	5.41	0.023	-263.38	
*Forehead - weighted*						
Median Shannon	0.038	0.090	32.2	3.61 e -7	-190.67	0.580
Gender	0.023	0.027	9.54	0.003	-193.54	
Roommates (n)	0.039	0.016	5.70	0.02	-196.16	
*Gut - unweighted*						
Median Shannon	0.063	0.081	73.24	4.3 e -12	-240.90	0.570
Over-the-counter acne product	0.014	0.013	11.18	0.001	-249.97	
University	0.014	0.007	6.64	0.012	-254.34	
*Gut - weighted*						
Median Shannon	0.107	0.238	20.64	2.61 e -5	-85.83	0.319
Over-the-counter acne product	0.034	0.065	5.65	0.021	-90.53	
University	0.028	0.047	4.08	0.047	-90.55	
*Palm - unweighted*						
Exercise frequency	0.033	0.022	15.74	2.00 e -4	-188.6	0.310
Lives with dogs	0.014	0.010	7.18	0.009	-189.9	
Roommates (n)	0.016	0.008	5.77	0.019	-191.8	
*Palm - weighted*						
Antibiotic use	0.026	0.024	4.97	0.029	-129.8	0.080
*Tongue - unweighted*						
Antibiotic use	0.018	0.015	7.75	0.007	-217.82	0.215
Median Shannon	0.038	0.010	5.5	0.022	-220.12	
*Tongue - weighted*						
No good model						

Generalized linear models (GLMs) were used to determine which of the measured factors or combination of factors best predicted variability in microbiome membership (unweighted UniFrac) and structure (weighted UniFrac). Unweighted UniFrac distances are a metric of the phylogenetic dissimilarity of samples through time. Weighted UniFrac distances weight dissimilarity both as a function of the phylogenetic dissimilarity and the relative abundance of taxa (such that two samples with the same phylogenetic dissimilarity are considered more different if one is dominated by a particular taxon).

BIC = Bayesian Information Criterion.

**Figure 3:**
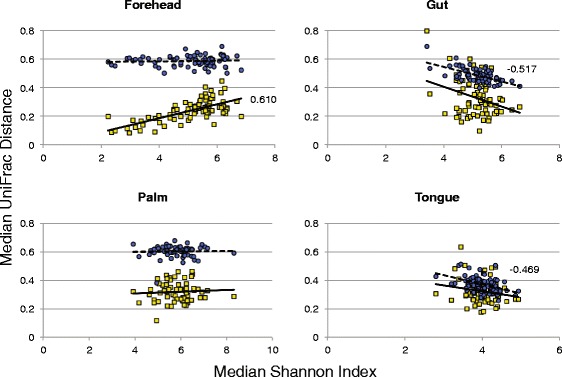
**Relationship between diversity and variability of microbial communities associated with each body habitat.** Diversity was measured as the median Shannon Diversity Index for each individual over the 3-month sampling period. Variability was measured as intra-individual median weighted (white boxes) and unweighted (gray circles) UniFrac distance. Each point represents values of the time-series data for one individual. Spearman rank correlation coefficients are presented for statistically significant relationships (*P* ≤0.01). Note that similar patterns were observed with other alpha diversity metrics (Additional file 11).

To explore the relationship between diversity and temporal variability in greater detail, we generated single-factor linear models using median Shannon index values as our metric of diversity and either median weighted or unweighted UniFrac values as our metric of stability (Figure 3). With these models, we observed statistically significant negative correlations between diversity and compositional variability for the gut and tongue communities; individuals with more diverse communities were less variable (more stable) than individuals with less diverse communities. In contrast, a positive relationship was observed between forehead community diversity and structural variability while no relationship was evident for palm communities. Similar directional patterns were observed with the other diversity metrics (Additional file 11). Our finding that microbial communities which likely experience lower rates of colonization from external environments (the gut and tongue) exhibit a positive diversity-stability relationship parallels patterns observed in many plant and animal communities where increases in species diversity often result in more stable communities and communities that are more resistant to invasions (that is, the portfolio effect) [22]. Although the health implications of the diversity-stability relationships observed here remain undetermined, recent work has shown that gut communities of lower diversity are often associated with disease phenotypes in humans [23].

**Figure 4:**
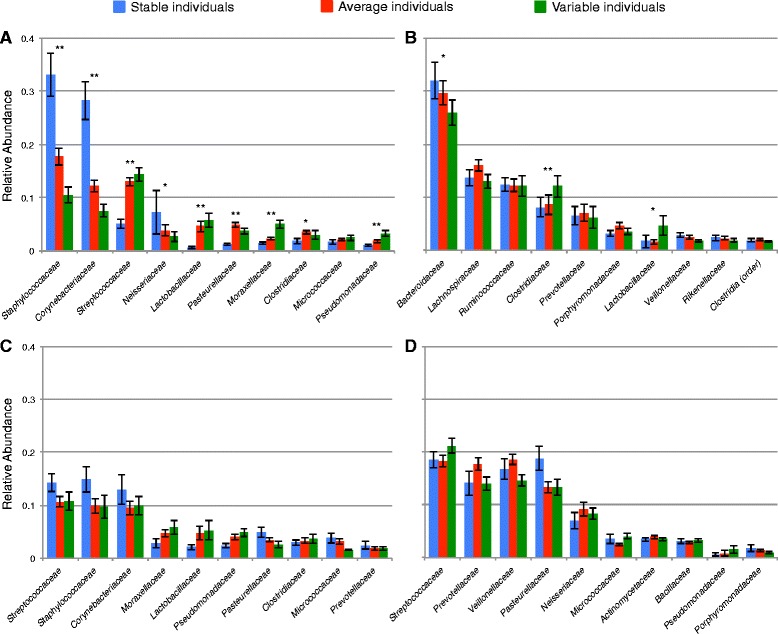
**Average taxonomic composition was different among stability classes across individuals.** Individuals were assigned to stability classes based on quartiles (first = stable (blue), second and third = average (red), fourth = variable (green)) of median weighted UniFrac distances for each body habitat. Significant differences were observed across forehead **(A)** and gut **(B)** communities but not in palm **(C)** or tongue **(D)** communities as determined by rank transforming the most abundant bacterial families (>1% in any group) for each body habitat and testing for differences between stability classes using the nonparametric Kruskal-Wallis analysis of variance. Significance is denoted with asterisks (* = corrected *P* ≤0.05, ** = corrected *P* ≤0.01).

Individuals that had more stable communities harbored taxonomically distinct communities compared with those found in more variable individuals (Figure 4). For example, individuals with stable forehead communities had a greater relative abundance of *Staphylococcaceae* and *Corynebacteriaceae*, whereas individuals with highly variable forehead communities were enriched in *Streptococcaceae* and *Lactobacillaceae* (Figure 4a). The trade-off between *Staphylococcaceae* and *Lactobacillaceae* is intriguing because several *Lactobacillaceae* species inhibit attachment of *Staphylococcaceae* to epithelial cells [24,25]. In the gut, two of the dominant groups of Firmicutes, *Clostridiaceae* and *Lactobacillaceae*, were more abundant in variable individuals, whereas the *Bacteroidaceae* (the dominant family within the Bacteroidetes phylum) were most abundant in stable individuals (Figure 4b). A higher Firmicutes:Bacteriodetes ratio has been observed in guts of obese individuals [26,27], but we did not have enough diversity in body mass index (BMI) to formally test if temporal variability may also be associated with obesity. Although the mechanisms underlying these patterns remain unclear, these observations highlight the likely importance of bacterial interactions in determining the stability of human-associated microbial communities.

## Conclusions

Our findings suggest that the high degree of temporal variability in alpha diversity levels, community membership, and community structure observed across the sampled body habitats and across study participants is important to consider when designing studies to assess linkages between the human microbiome and health. Although the variability in community composition among healthy individuals exceeds the variability within individuals over time (Additional file 6, [3,5,8]), the intra-individual temporal variability is considerable and the degree of variability that an individual experiences over time may be a factor in determining disease state or differential treatment success. Further, because variability through time can be high, samples collected at one point in time may not adequately characterize an individual's microbiome, even if focusing on only the more abundant phylotypes (Figure 1b, dark shades). If the effect size of a change in disease state on the human microbiome is sufficiently large (for example, the loss of a major lineage), this intra-individual temporal variability may be irrelevant. However, if changes in disease state are associated with more subtle shifts in microbial community composition, it would be important to control for this temporal variability before one could establish causal linkages between changes in the microbiome and changes in health status. It is now well established that there is considerable inter-individual variability in the composition of the human microbiome [5,18], leading to the concept of a ‘personal microbiome’, and we are beginning to establish causal relationships between composition of the microbiome and disease [28]. Here we show that there is also a high-degree of inter-individual variability in the stability of the human gut, tongue, forehead, and palm microbiome. As a result, we suggest that the ‘personal microbiome’ concept should be extended to incorporate the rate of change of an individual’s microbiome, in addition to its composition (a feature which distinguishes the ‘personal microbiome’ from the ‘personal genome’) and that future investigations into associations between features of the microbiome and host phenotype may want to consider temporal variability as a potential explanatory factor.

## Methods

### Subject recruitment and sample collection

Volunteers were recruited from three Universities (University of Colorado, Boulder (UCB), Northern Arizona University (NAU), and North Carolina State University (NCSU)) in January/February of 2012 and asked to donate weekly self-collected samples for a minimum of 10 weeks using sterile, pre-labeled, double-tipped swabs (Becton, Dickinson and Company, Sparks, MD, USA.). Participants were instructed to sample two skin habitats (foreheads and palms) and the surface of their tongue by swabbing for 10 to 15 s. Gut (fecal) samples were collected by touching cotton swabs to used toilet paper so that a small amount of fecal material was transferred to each pair of swabs. Volunteers were asked to collect samples before showering and as close to drop-off times as possible without placing samples in freezers to avoid freeze-thaw cycles. One representative at each University collected samples from students and placed them in a -20°C freezer until shipping on dry ice to the UCB where all sample processing occurred. Volunteers were also asked to provide a variety of demographic and behavioral metadata at the initiation of the project using a scantron-based survey (Additional file 1). Weekly questionnaires (Additional file 2) were also provided with sampling kits to collect information on changes in health status, medication use, and menstruation for women. At the conclusion of the study prior to publication, study participants were provided their personalized results via a password-protected website [29]. All volunteers were made aware of the nature of this project and gave written consent in accordance with protocols approved by each University's Institutional Review Board (IRB) (UCB 409.13; NAU 12.0169; NCSU 2443). Per IRB regulations, volunteers were able to drop out of the study at any time and were not required to answer any or all survey questions.

### Sample processing

Samples from NAU and NCSU were shipped on dry ice to UCB at the conclusion of sampling. Upon arrival, individual swabs were linked with Personal IDs using digital barcodes and logged into an Excel worksheet. Swabs were then sorted by body habitat and the tip of one duplicate swab was aseptically cut into single wells in 2 mL 96-well deep-well plates (Axygen Inc., Union City, CA, USA). Plates were sealed with silicone Axymat sealing mats (Axygen Inc., Union City, CA, USA). Each plate contained negative control samples that included swab blanks (sterile swabs), extraction blanks (reagents), and a PCR control. Forehead, gut, and tongue plates also included positive controls that were collected from one individual at the initiation of the project and stored/shipped with samples at each university. No differences were observed in community membership or structure in positive control samples.

### DNA extraction, PCR amplification, and sequencing

DNA extraction and PCR amplification of the variable region 4 (V4) of the 16S rRNA gene using Illumina adapted universal primers 515 F/806R [12,30] was conducted using the direct PCR protocol as previously described [31].

Aliquots (4 μL) from the fecal and tongue extracts were transferred into 384-well plates for triplicate PCR reactions, while skin aliquots (forehead and palm, 4 μL) were transferred into 96-well plates. PCRs were conducted in triplicate 20 μL reactions and thermal cycling conditions for the 384-well plates were: initial denaturation for 3 min at 94°C; 35 cycles (94°C, 60 s; 50°C, 60 s; 72°C, 105 s) followed by a final elongation for 10 min at 72°C. Conditions for the 96-well plates were identical except for shorter denaturation (94°C, 45 s) and elongation (72°C, 90 s) steps. PCR products from triplicate reactions of each sample were pooled, visualized on an agarose gel, and quantified using the PicoGreen dsDNA assay (Invitrogen, Carlsbad, CA, USA). Positive amplicons from each body habitat (forehead, gut, palm, and tongue) were then pooled in equimolar concentrations into composite samples that were cleaned using a single-tube MoBio Ultraclean PCR Clean-up Kit (MoBio Laboratories, Carlsbad, CA, USA). Each body habitat was sequenced on an individual lane (4 lanes total) of an Illumina HiSeq2000 instrument at the University of Colorado BioFrontiers Institute Advanced Genomics Facility.

### Data processing

All data processing was performed using QIIME 1.6.0-dev unless otherwise noted. The specific processing steps were as follows. Raw fastq data were demultiplexed and quality filtered as described previously [32]. Sequences that passed quality filtering were clustered into phylotypes (Operational Taxonomic Units, OTUs) at 97% sequence identity using a uclust-based [33] closed-reference protocol against the 12_10 revision of the Greengenes database [34], where reads that did not match a sequence in the reference set at least 97% identity were excluded from subsequent analyses. The taxonomy of each phylotype was assigned as the taxonomy associated with the Greengenes sequence defining that OTU. The Greengenes phylogenetic tree was used for phylogenetic diversity calculations. A median of 49,242.0 sequences was collected per sample. After removing phylotypes appearing in negative controls at high abundance (≥0.5% across all controls) [31], all samples were rarefied to 10,000 sequences for all downstream analyses unless otherwise noted.

Potentially mislabeled samples were detected using the random forest classification approach described previously [35]. Briefly, the full sample-by-phylotype abundance matrix (that is, OTU table) was filtered to exclude phylotypes that were observed in fewer than 10 samples. The OTU table was then randomly subsampled to exactly 1,000 sequences per sample. Three samples achieved a probability of being mislabeled greater than 90%, and were excluded from all downstream analyses.

Alpha diversity metrics (phylogenetic diversity (PD), phylotype richness, and Shannon Index [13]) were computed as implemented in QIIME. Comparisons of alpha diversity presented in this study are computed at exactly 10,000 sequences per sample. Beta diversity was computed using the weighted and unweighted UniFrac metrics [16] at exactly 10,000 sequences per sample.

The time series samples were defined as the set of samples that came from an individual’s body site where at least seven samples were collected and successfully sequenced from that individual’s body site over a 10-week sampling period. For example, if six fecal samples and seven forehead samples were sequenced from an individual, their fecal samples would not be included in any time series analyses, but their forehead samples would be included. This resulted in 80 individuals for which we had a forehead time series (48 women, 30 men, 2 unknown), 75 individuals with a gut time series (48 women, 26 men, 1 unknown), 61 individuals with a palm time series (35 women, 25 men, 1 unknown), and 80 individuals with a tongue time series (50 women, 28 men, 2 unknown) from 85 subjects (Table 1).

All QIIME commands for performing these processing steps can be found in Additional file 12.

### Statistical analysis

To assess the temporal variability of within sample diversity (alpha diversity), we calculated the coefficient of variation (CV) for three diversity metrics (phylogenetic diversity - PD, OTU richness, and Shannon index) for each body habitat of each individual through time. Individual values were used to determine the per body site median across the study population, with higher values indicative of more variable communities.

Variability in community composition (beta diversity) was determined per body habitat by calculating the median weighted and unweighted UniFrac distances for each individual over time. With this metric, communities with a higher median value are more variable whereas a lower value indicates more stable communities. (Note that because we summarize temporal data in a single measurement, we do not need to account for lack of independence of temporal samples from a single individual in evaluations based on this metric.) Differences across body sites for both alpha- and beta-diversity were assessed using the non-parametric Kruskal-Wallis one-way analysis of variance with pairwise comparisons made using the Mann-Whitney *U*-test, as implemented in R.

To determine the number of phylotypes shared by an individual over different windows of time, we converted the OTU tables of each body habitat to a presence/absence matrix, split it by individual, filtered out singletons, and determined the number of OTUs found in exactly two samples, three samples, four samples, and so on up to seven samples using a custom R script. Samples did not have to be from consecutive weeks. We repeated this analysis on only the top 10% most abundant OTUs per individual. The numbers of phylotypes shared per individual were then averaged across individuals for each window of time and each body habitat.

For each body habitat, the study population was divided into quartiles based on median intra-individual UniFrac values where the first quartile was defined as ‘stable’, the second and third quartiles as ‘average’, and the fourth quartile as ‘variable.’ To determine if certain taxa were more or less abundant in the different quartiles (that is, stability classes), we rank transformed the most abundant bacterial families (>1% across individuals) for each body habitat and tested for differences between the groups using the nonparametric Kruskal-Wallis analysis of variance.

adonis [36], ANOSIM [37], and PERMDISP [36] (using 999 permutations) were used to test for differences in community composition between individuals at each body site. The statistical methods were used to analyze both weighted and unweighted UniFrac distance matrices, with only the time series samples being included in the analyses.

To determine the affect of antibiotic use on community variability, we grouped individuals based on their usage (yes or no) and used the non-parametric Mann-Whitney *U*-test to test for differences between the two groups. Spearman rank correlations were used to determine if community variability as measured using median UniFrac distances was correlated across pairs of body habitats. To assess if patterns in community composition could be related to time between sampling events, Mantel tests (Spearman-rank correlations on 999 permutations) were conducted for each individual using both weighted and unweighted UniFrac values and Manhattan time-distance matrices calculated in R using the VEGAN package [35]. Using the mean of the different alpha diversity metrics (PD, phylotype richness, and Shannon index [13]) as our metrics of diversity and median UniFrac distances (both weighted and unweighted) as our metric of community variability on a per individual basis, we constructed linear models for each body habitat across individuals to examine the relationship between diversity and stability.

We identified key predictors of the variability in composition of bacterial assemblages using generalized linear models. We used a model simplification procedure, removing non-significant terms (*α* =0.05) in a stepwise fashion [38], to explore the relative contributions of the various terms included in the start model. Model simplification approaches have been criticized [39], but in the absence of strong *a priori* information on the drivers of variability of bacterial assemblages, this approach is a useful first step [40]. The final models we present are those that exclusively include variables that explain significant variation in our dependent variables. We also used model simplification in which final models were those in which Bayesian information criterion (BIC) was minimized. However, these ‘best’ models ended up including all variables we tested and so here we focus on those variables with significant explanatory power.

To determine if the weeks where individuals reported taking antibiotics were the weeks where they experience the largest changes in their gut community compositions, we ran per-body-site one-tailed, rank-based Monte Carlo t-tests. The adjacent-week UniFrac distances were compiled for each individual on a per-body-site basis (that is, the distance between their gut samples on week 1 and week 2, week 2 and week 3, and so on). Each individual’s UniFrac distances were ranked from smallest to largest, and assigned their rank value. Across individual ranks were grouped into distributions based on whether they occurred in a week where the individual reported taking antibiotics or not. Those distributions were then compared with a one-tailed Monte Carlo *t*-test with 1,000 iterations.

### Data availability

Sequence data and accompanying de-identified metadata have been deposited in the EMBL under accession number (ERP005150-ERP005153).

## Additional files

Additional file 1:
**The pre-study questionnaire used to collect demographic, lifestyle, and hygiene data on study participants.**
Additional file 2:
**The weekly questionnaire used to collect information about changes in health status, medication use, stage of menstrual cycle for women, and any other dramatic changes in the routine of study participants.**
Additional file 3:
**A list of all samples collected in this study with corresponding de-identified personal IDs of study subjects and answers to survey questions.**
Additional file 4:
**A figure showing the amount of microbial diversity observed in each sample.**
Additional file 5:
**A figure depicting the temporal variability observed in microbial community membership and structure for each body habitat of each individual.**
Additional file 6:
**A table showing that the composition of each individual’s microbiome is personalized through time.**
Additional file 7:
**A figure showing how the microbial communities of selected individuals vary through time.**
Additional file 8:
**A table showing the results of Spearman rank correlation of community membership across different body habitats.**
Additional file 9:
**A table showing the results of Spearman rank correlation of community structure across different body habitats.**
Additional file 10:
**A table of Mantel test results correlating microbial community membership and structure with time between samples (time distance-decay).**
Additional file 11:
**A table of results correlating microbial diversity with temporal variability in community membership and structure for each body habitat.**
Additional file 12:
**A list of all QIIME commands used in data processing.**

## Abbreviations

BIC: Bayesian information criterion
BMI: body mass index
CA: California
CV: coefficient of variation
dsDNA: double-stranded deoxyribonucleic acid
GLM: generalized linear model
IRB: Institutional Review Board
MD: Maryland
mL: milliliter
NAU: Northern Arizona University
NCSU: North Carolina State University
OTU: operational taxonomic unit
PCR: polymerase chain reaction
PD: phylogenetic diversity
QIIME: Quantitative Insights Into Microbial Ecology
rRNA: ribosomal ribonucleic acid
sec: seconds
UCB: University of Colorado, Boulder
μL: microliter
U.S.: United States
USA: United States of America
V4: variable region 4
