# Supplementary material for: Temporal variability is a personalized feature of the human microbiome
Source: Genome Biol. 2014 Dec 3;15(12):531. doi: 10.1186/s13059-014-0531-y (PMC4252997; doi:10.1186/s13059-014-0531-y)
Supplement: Additional file 1: — The pre-study questionnaire used to collect demographic, lifestyle, and hygiene data on study participants. [file 13059_2014_531_MOESM1_ESM.pdf]

Explaining variability among healthy human-associated microbial communities

GENERAL QUESTIONNAIRE

<u>You may decline to answer any question by leaving the space blank.</u>

Please fill out your scantron with your name, gender, date of birth, height, weight and Zip Code of residence in the areas indicated below. In this example, John G. Doe is the respondent. He is a male, living in Zip Code 80302, was born on April 15, 1989, and is 5'9" tall and weighs 180 pounds.

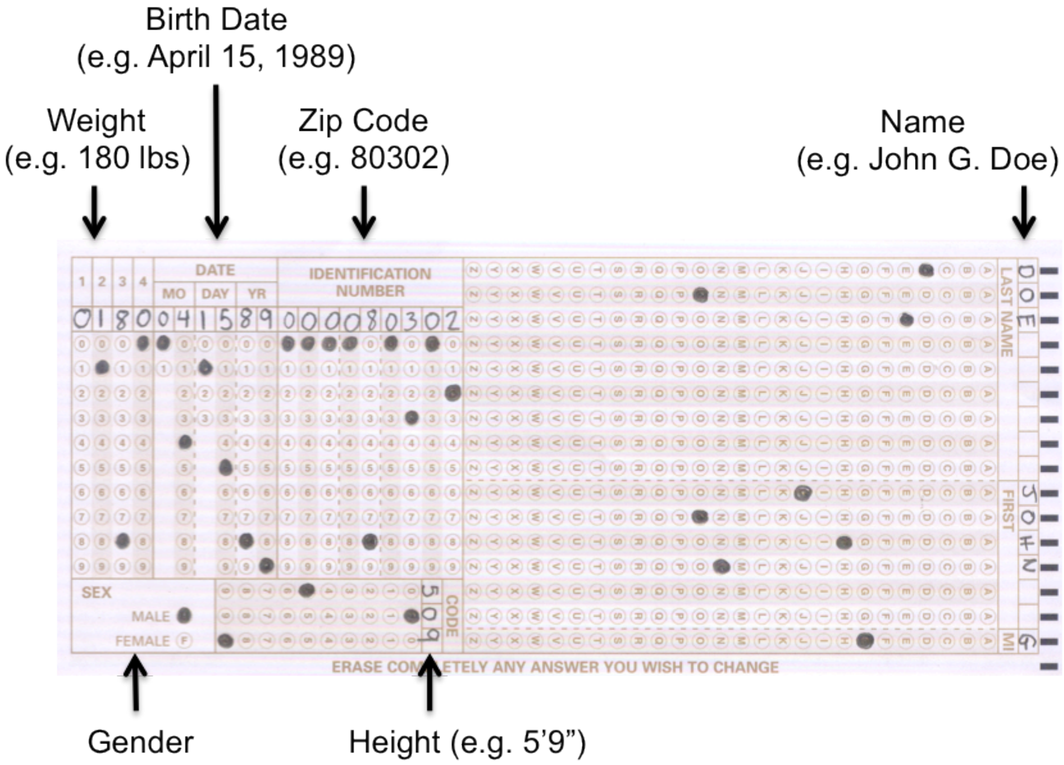

Please answer the questions below by filling out the appropriate answer on the accompanying scantron form.

General Diet Information

1.) How would you classify your diet?
   a. Omnivore
   b. Omnivore but do not eat red meat
   c. Vegetarian
   d. Vegetarian but eat seafood
   e. Vegan

2.) Are you taking a daily multivitamin?
   a. Yes
   b. No

3.) Are you taking any other nutritional/herbal supplements?
   a. Yes**, *if yes please indicate what you are taking in the appropriate space on your scantron form*.
   b. No

4.) Are you lactose intolerant?
   a. Yes
   b. No

5.) Are you gluten intolerant?
   a. Yes
   b. No

6.) I am allergic to __________. (mark all that apply)
   a. Peanuts
   b. Tree nuts
   c. Shellfish
   d. Other
   e. I have no food allergies that I know of.

7.) Do you have diabetes?
   a. Yes, Type 1
   b. Yes, Type 2
   c. No

8.) Do you follow any other special diet restrictions other than those indicated above?
   a. Yes**, *if yes please explain in the appropriate space on your scantron form*.
   b. No

General Information

9.) What is your race/ethnicity?
   a. Caucasian
   b. Asian or Pacific Islander
   c. African American
   d. Hispanic
   e. Other**, *please explain in the appropriate space on your scantron form.*

10.) When did you move to Colorado (Arizona, North Carolina)?
   a. Within the past month
   b. Within the past 3 months
   c. Within the past 6 months
   d. Within the past year
   e. I have lived in Colorado (Arizona, North Carolina) for more than a year.

11.) I have traveled outside of the United States in the past _________.
   a. Month
   b. 3 months
   c. 6 months
   d. 1 year
   e. I have not been outside of the United States in the past year.

12.) Do you live in on-campus housing?
   a. Yes
   b. No
13.) Do you live in a fraternity/sorority house?
   a. Yes
   b. No
14.) Do you eat the majority of your meals in campus dining halls?
   a. Yes
   b. No
15.) How many roommates do you have?
   a. None
   b. One
   c. Two
   d. Three
   e. More than three
16.) Do you have a dog(s)?
   a. Yes
   b. No
17.) Do you have a cat(s)?
   a. Yes
   b. No
18.) Which is your dominant hand?
   a. I am right handed
   b. I am left handed
   c. I am ambidextrous

General Lifestyle and Hygiene Information

19.) Do you use any artificial tanning methods?
   a. Yes – tanning beds
   b. Yes – tanning sprays
   c. No
20.) How often do you exercise?
   a. Daily
   b. Regularly (3-5 times/week)
   c. Occasionally (1-2 times/week)
   d. Rarely (few times/month)
   e. Never
21.) Do you generally exercise indoors or outdoors?
   a. Indoors
   b. Outdoors
   c. Both
   d. Depends on the season
   e. None of the above
22.) Do you bite your fingernails?
   a. Yes
   b. No

23.) How often do you use a swimming pool/hot tub?
   a. Daily
   b. Regularly (3-5 times/week)
   c. Occasionally (1-2 times/week)
   d. Rarely (few times/month)
   e. Never
24.) How often do you smoke cigarettes?
   a. Daily
   b. Regularly (3-5 times/week)
   c. Occasionally (1-2 times/week)
   d. Rarely (few times/month)
   e. Never
25.) How often do you drink alcohol?
   a. Daily
   b. Regularly (3-5 times/week)
   c. Occasionally (1-2 times/week)
   d. Rarely (few times/month)
   e. Never
26.) How often do you brush your teeth?
   a. Daily
   b. Regularly (3-5 times/week)
   c. Occasionally (1-2 times/week)
   d. Rarely (few times/month)
   e. Never
27.) How often do you floss your teeth?
   a. Daily
   b. Regularly (3-5 times/week)
   c. Occasionally (1-2 times/week)
   d. Rarely (few times/month)
   e. Never
28.) How often do you wear facial cosmetics?
   a. Daily
   b. Regularly (3-5 times/week)
   c. Occasionally (1-2 times/week)
   d. Rarely (few times/month)
   e. Never
29.) Do you use deodorant or antiperspirant (antiperspirants generally contain aluminum)?
   a. I use deodorant
   b. I use an antiperspirant
   c. Not sure, but I use some form of deodorant/antiperspirant
   d. I do not use deodorant or an antiperspirant
30.) Approximately how many hours of sleep to you get in an average night?
   a. Less than 5 hours
   b. 5-6 hours
   c. 6-7 hours
   d. 7-8 hours
   e. 8 or more hours

31.) Do you use fabric softener when drying your clothes?
   a. Yes
   b. No

General Health Information

32.) I have taken antibiotics in the last ____________.
   a. Week
   b. Month
   c. 6 months
   d. Year
   e. I have not taken antibiotics in the past year.

33.) I have gotten a flu vaccine in the last ____________.
   a. Week
   b. Month
   c. 6 months
   d. Year
   e. I have not gotten the flu vaccine in the past year.

34.) Are you currently using some form of hormonal birth control?
   a. Yes, I am taking the "pill"
   b. Yes, I use an injected contraceptive (DMPA)
   c. Yes, I use a contraceptive patch (Ortho-Evra)
   d. Yes, I use the NuvaRing
   e. No

35.) Are you currently pregnant?
   a. Yes**, *if yes please indicate your due date in the appropriate space on your scantron form*.
   b. No
   c. Not sure

36.) My weight has _________ within the last 6 months.
   a. Increased more than 10 pounds
   b. Decreased more than 10 pounds
   c. Remained stable

37.) Have you had your tonsils removed?
   a. Yes
   b. No

38.) Have you had you appendix removed?
   a. Yes
   b. No

39.) Have you had chickenpox?
   a. Yes
   b. No

40.) Do you currently take prescription medication for facial acne?
   a. Yes
   b. No

41.) Do you use over the counter products to control facial acne?
    a. Yes
    b. No
42.) Do you currently take over the counter or prescription medication for other conditions?
    a. Yes**, *if yes please explain in the appropriate space on your scantron form*.
    b. No
43.) Were you born via cesarean section (C-section)?
    a. Yes
    b. No
    c. Not sure
44.) Do you have phenylketonuria (PKU)?
    a. Yes
    b. No
45.) Do you have asthma?
    a. Yes
    b. No
46.) Do you have seasonal allergies?
    a. Yes
    b. No
47.) Do you have any of the following non-food allergies? (mark all that apply)
    a. Drug (e.g. Penicillin)
    b. Pet dander
    c. Beestings
    d. Poison ivy/oak
    e. Sun
48.) I have Inflammatory Bowel Disease in the form of __________.
    a. Crohn's disease
    b. Colitis
    c. I do not have inflammatory bowel disease.
49.) I have a skin condition in the form of ___________.
    a. Eczema
    b. Psoriasis
    c. Rosacea
    d. I do not have a skin condition.

On the blank section on the back of your scantron form, please write anything else about yourself that you think might affect your personal microorganisms (this information may be published, but not in an identifying manner):
