# Supplementary material for: Temporal variability is a personalized feature of the human microbiome
Source: Genome Biol. 2014 Dec 3;15(12):531. doi: 10.1186/s13059-014-0531-y (PMC4252997; doi:10.1186/s13059-014-0531-y)
Supplement: Additional file 2: — The weekly questionnaire used to collect information about changes in health status, medication use, stage of menstrual cycle for women, and any other dramatic changes in the routine of study participants. [file 13059_2014_531_MOESM2_ESM.pdf]

Explaining variability among healthy human-associated microbial communities

WEEKLY QUESTIONNAIRE

<u>You may decline to answer any question by leaving the space blank.</u>

1.) Have you been sick since the last sampling day?
  a. Yes
  b. No
2.) If you answered yes to Question 1 above, please circle <u>all symptoms</u> that apply.
  a. Stomach ache
  b. Fever
  c. Vomiting
  d. Cold sweats
  e. Body aches
  f. Headache
  g. Runny nose
  h. Congestion
  i. Coughing
3.) Have you taken any antibiotics since the last sampling day?
  a. Yes**
  b. No

  ***If yes, please indicate which antibiotic you took and what you were treating with the antibiotics in the space provided.*

4.) Women, are you currently or have you menstruated since the last sampling day?
  a. Yes, I am currently menstruating
  b. Yes, I menstruated since the last sampling day but am not currently
  c. No, I am not and have not menstruated since the last sampling day
  d. No, I am taking birth control that does not permit me to menstruate
  e. No, I am pregnant

5.) Have there been any other changes in your routine activities (e.g. changed diet, quit smoking, moved residence, got flu vaccine etc.) that you think could influence the microbial communities associated with your various body habitats? *Please use the space below and/or back of this sheet to answer.*
