# Supplementary figures and images for: Temporal variability is a personalized feature of the human microbiome
Source: Genome Biol. 2014 Dec 3;15(12):531. doi: 10.1186/s13059-014-0531-y (PMC4252997; doi:10.1186/s13059-014-0531-y)

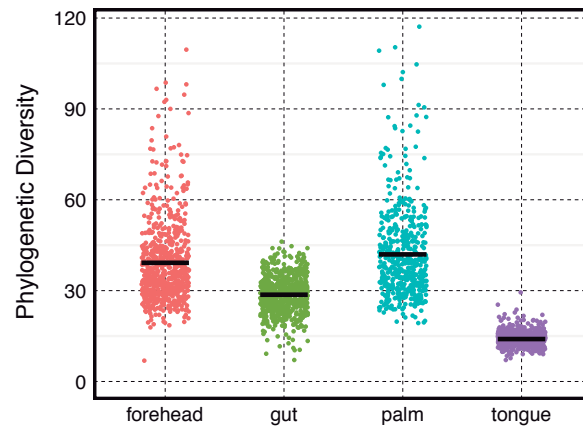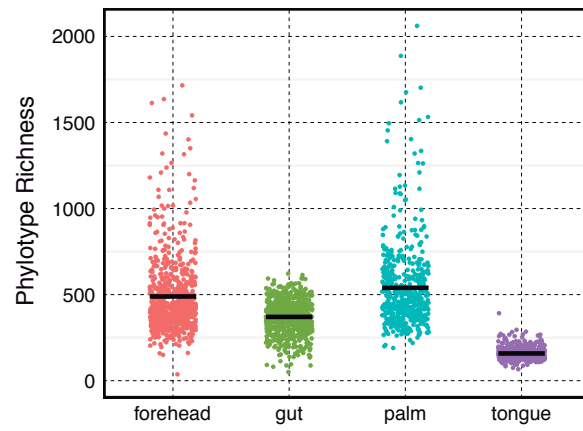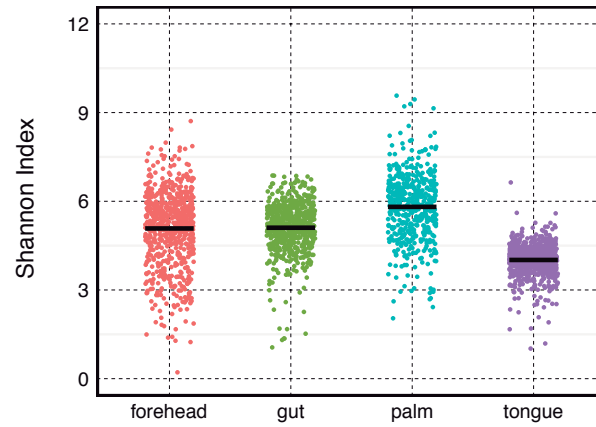

Supplement: Additional file 4: — A figure showing the amount of microbial diversity observed in each sample. [file 13059_2014_531_MOESM4_ESM.pdf]

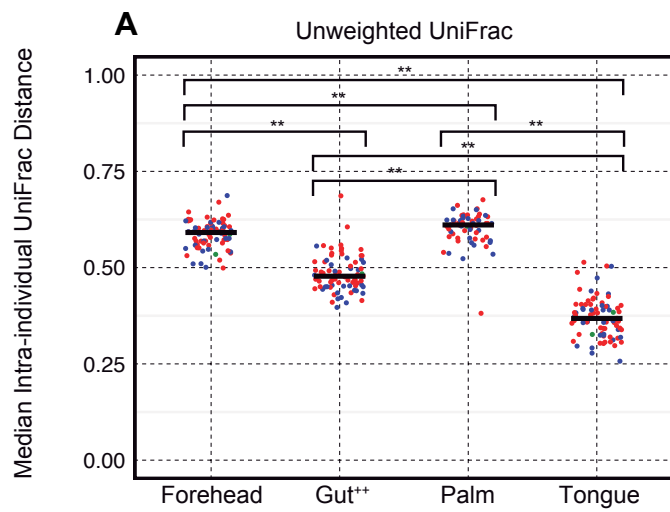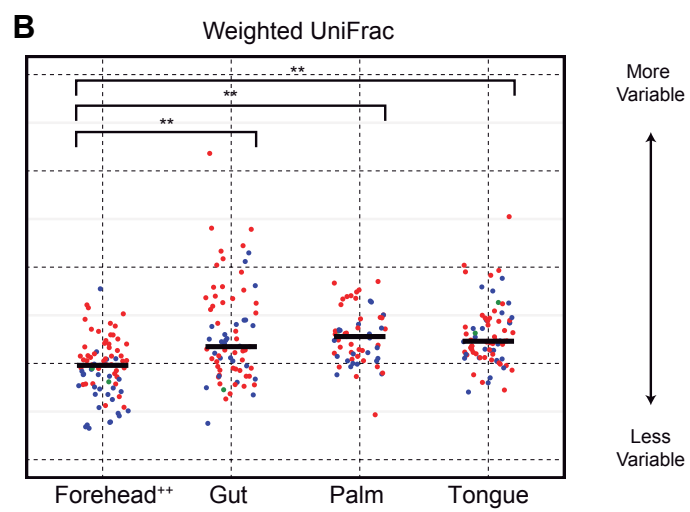

Supplement: Additional file 5: — A figure depicting the temporal variability observed in microbial community membership and structure for each body habitat of each individual. [file 13059_2014_531_MOESM5_ESM.pdf]

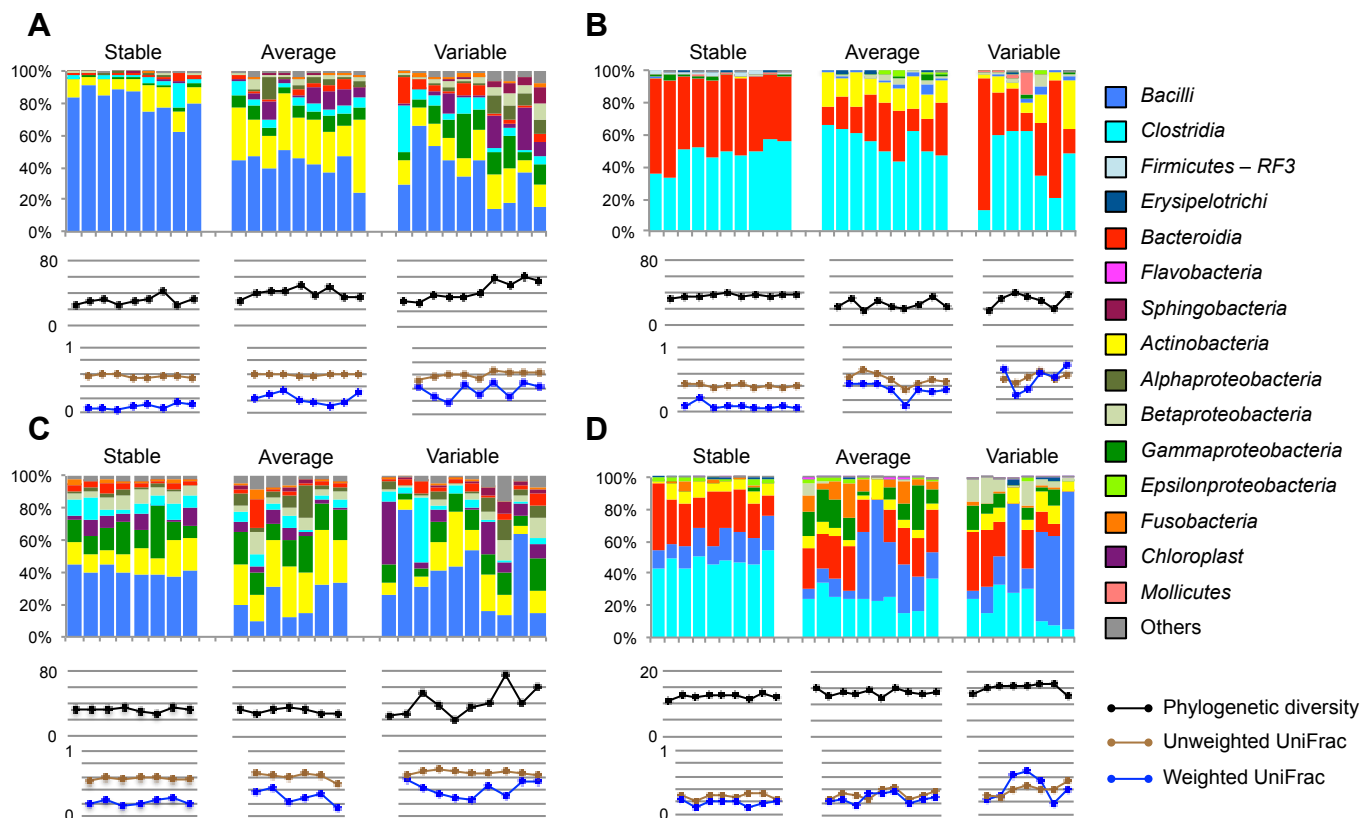

Supplement: Additional file 7: — A figure showing how the microbial communities of selected individuals vary through time. [file 13059_2014_531_MOESM7_ESM.pdf]
