# Supplementary material for: Temporal variability is a personalized feature of the human microbiome
Source: Genome Biol. 2014 Dec 3;15(12):531. doi: 10.1186/s13059-014-0531-y (PMC4252997; doi:10.1186/s13059-014-0531-y)
Supplement: Additional file 6: — A table showing that the composition of each individual’s microbiome is personalized through time. [file 13059_2014_531_MOESM6_ESM.pdf]

Additional file 6. The composition of each individual's microbiome was unique as determined by three different non-parametric, permutational tests. Test statistics and p-values are reported for each comparison.

	Adonis (R^2)	ANOSIM (R)	PERMDISP (F)
**Forehead**			
Weighted UniFrac	0.622; 0.001	0.637; 0.001	3.653; 0.001
Unweighted UniFrac	0.293; 0.001	0.678; 0.001	3.625; 0.001
**Gut**			
Weighted UniFrac	0.444; 0.001	0.428; 0.001	3.140; 0.001
Unweighted UniFrac	0.485; 0.001	0.860; 0.001	2.522; 0.001
**Palm**			
Weighted UniFrac	0.436; 0.001	0.433; 0.001	1.665; 0.004
Unweighted UniFrac	0.288; 0.001	0.651; 0.001	3.505; 0.001
**Tongue**			
Weighted UniFrac	0.467; 0.001	0.420; 0.001	1.843; 0.001
Unweighted UniFrac	0.365; 0.001	0.534; 0.001	3.227; 0.001
