# Supplementary material for: Temporal variability is a personalized feature of the human microbiome
Source: Genome Biol. 2014 Dec 3;15(12):531. doi: 10.1186/s13059-014-0531-y (PMC4252997; doi:10.1186/s13059-014-0531-y)
Supplement: Additional file 9: — A table showing the results of Spearman rank correlation of community structure across different body habitats. [file 13059_2014_531_MOESM9_ESM.pdf]

Additional file 9. Spearman rank correlations of median weighted UniFrac distances per individual across body habitats. Rho values and the number of individuals compared in each test are shown. ** = corrected $p \leq 0.01$.

	Forehead	Gut	Palm
Forehead			
Gut	-0.076; n=71		
Palm	0.413**; n=61	-0.138; n=55	
Tongue	0.173; n=76	0.232; n=71	0.013; n=58
