# Supplementary material for: Temporal variability is a personalized feature of the human microbiome
Source: Genome Biol. 2014 Dec 3;15(12):531. doi: 10.1186/s13059-014-0531-y (PMC4252997; doi:10.1186/s13059-014-0531-y)
Supplement: Additional file 10: — A table of Mantel test results correlating microbial community membership and structure with time between samples (time distance-decay). [file 13059_2014_531_MOESM10_ESM.pdf]

Additional file 10. Summary of Mantel tests of correlation between time distance measure in weeks and community composition. Numerator of fraction represents the number of individuals with a statistically significant positive correlation (uncorrected $p \leq 0.05$) based on Spearman's rank correlation. Denominator is total number of individuals compared. Note that these results are based on uncorrected p-values and the expected number of false positives rounded to the nearest whole number is indicated in parentheses.

	Forehead	Gut	Palm	Tongue
Weighted UniFrac	8/80<br>(4)	7/75<br>(4)	7/61<br>(3)	15/80<br>(4)
Unweighted UniFrac	20/80<br>(4)	12/75<br>(4)	13/61<br>(3)	14/80<br>(4)
