# Supplementary material for: Temporal variability is a personalized feature of the human microbiome
Source: Genome Biol. 2014 Dec 3;15(12):531. doi: 10.1186/s13059-014-0531-y (PMC4252997; doi:10.1186/s13059-014-0531-y)
Supplement: Additional file 11: — A table of results correlating microbial diversity with temporal variability in community membership and structure for each body habitat. [file 13059_2014_531_MOESM11_ESM.pdf]

Additional file 11. Relationship between diversity and stability for microbial communities associated with four human body habitats. Spearman's rho values were determined for each combination of median weighted (WUniFrac) and unweighted (UUniFrac) UniFrac distances and median diversity values over time across individuals. Positive rho values indicate that more diverse communities are less stable over time while negative values indicate that more diverse communities are more stable. Statistical significance is indicated with asterisks (* = uncorrected $p \leq 0.05$, ** = uncorrected $p \leq 0.01$).

	Forehead		Gut		Palm		Tongue	
	WUniFrac	UUniFrac	WUniFrac	UUniFrac	WUniFrac	UUniFrac	WUniFrac	UUniFrac
Median Shannon	0.610**	0.064	-0.168	-0.517**	0.051	-0.088	-0.152	-0.469**
Median PD	0.267*	0.074	-0.185	-0.579**	-0.046	-0.164	-0.055	-0.125
Median Richness	0.292**	0.042	-0.343**	-0.624**	-0.094	-0.192	-0.096	-0.007
